# The Impact of Interleukin 28b Gene Polymorphism on the Virological Response to Combined Pegylated Interferon and Ribavirin Therapy in Chronic HCV Genotype 4 Infected Egyptian Patients Using Data Mining Analysis

**DOI:** 10.5812/hepatmon.10509

**Published:** 2013-07-17

**Authors:** Marwa Khairy, Rabab Fouad, Mahassen Mabrouk, Wafaa El-Akel, Abu Bakr Awad, Rabab Salama, Mayada Elnegouly, Olfat Shaker

**Affiliations:** 1 Endemic Medicine Department and Hepatology Unit, Faculty of Medicine, Cairo University, Cairo, Egypt; 2 Bioinformatic and Statistic Department, Faculty of Computer Sciences, Cairo University, Cairo, Egypt; 3 Medical Biochemistry and Molecular Biology Department, Faculty of Medicine, Cairo University, Cairo, Egypt

**Keywords:** Hepatitis C Virus, IL28B Protein, Human, Decision Trees, Data Mining, Peginterferon Alfa-2a

## Abstract

Background: Chronic HCV represents one of the common causes of chronic liver disease worldwide with Egypt having the highest prevalence, namely genotype 4. Interleukin IL-28B gene polymorphism has been shown to relate to HCV treatment response, mainly in genotype1.

Objectives: We aim to evaluate the predictive power of the rs12979860 IL28B SNP and its protein for treatment response in genotype 4 Egyptian patients by regression analysis and decision tree analysis.

Patients and Methods: The study included 263 chronic HCV Egyptian patients receiving peg-interferon and ribavirin therapy. Patients were classified into 3 groups; non responders (83patients), relapsers (76patients) and sustained virological responders (104 patients). Serum IL 28 B was performed, DNA was extracted and analyzed by direct sequencing of the SNP rs 12979860 of IL28B gene.

Results: CT, CC and TT represented 56 %, 25 % and 19% of the patients, respectively. Absence of C allele (TT genotype) was significantly correlated with the early failure of response while CC was associated with sustained virological response. The decision tree showed that baseline alpha fetoprotein (AFP ≤ 2.68 ng/ml) was the variable of initial split (the strongest predictor of response) confirmed by regression analysis. Patients with TT genotype had the highest probability of failure of response.

Conclusions: Absence of the C allele was significantly associated with failure of response. The presence of C allele was associated with a favorable outcome. AFP is a strong baseline predictor of HCV treatment response. A decision tree model is useful for predicting the probability of response to therapy.

## 1. Background

Hepatitis C virus (HCV) infection is one of the main causes of chronic liver disease worldwide (1). Egypt has the highest prevalence of adult HCV infection in the world, averaging 15%-25% in rural communities (2), with > 90% of cases having HCV genotype 4 (3). Furthermore, epidemiological reports indicate that HCV genotype 4 is beginning to spread from its native African and Middle Eastern origins to countries of Southern Europe such as France, Italy and Spain and in some foci in the United States, particularly among intravenous drug users (4-6). Combined pegylated interferon and ribavirin therapy are still the most effective therapies in HCV-4 (7), although the rate of sustained virological response (SVR) is around 50% (8). It is of major interest for both patient care and economic approach to predict failure of response (9). Several independent genome-wide association studies (GWAS) reported single nucleotide polymorphisms (SNPs) near the IL28B (IFN-λ3) locus that displayed association with treatment response, mainly in HCV genotype 1 (10-12). There are a few data so far regarding the role of IL28B polymorphism in HCV-4 patients with respect to response to antiviral therapy or fibrosis progression (13). A recent study, investigated the levels of IL-29 and IL-28 in patients with different outcomes of HCV infection and demonstrated that patients with chronic hepatitis C had significantly lower IL-29 serum levels than subjects who had spontaneously cleared a previous HCV infection, healthy controls and carriers of a rs12979860 C allele consistently tended to have higher IL-29 and IL-28 serum levels than subjects with a T/T genotype in all study groups (14). The decision tree analysis tool, a core component of data mining analysis, was applied for the prediction of early virological response to combined interferon-ribavirin therapy in chronic HCV (15) and sustained virological response (16). Combined host factors such as age, gender, liver fibrosis in addition to laboratory investigations were identified as predictors of the therapeutic effects in chronic HCV patients through the application of data mining analysis (17).

## 2. Objectives

The objective of this study was to evaluate the predictive power of the rs12979860 IL28B SNP and its protein for treatment response in HCV Egyptian patients by regression analysis and decision tree analysis in relation to other predictors of response.

## 3. Patients and Methods

### 3.1. Selection of Patients

Cross sectional study included chronic HCV 263 patients candidate for treatment with combined interferon and ribavirin therapy. Patients were recruited from Quahera Fatemyia Hospital, a referral center for treatment of HCV in Egypt under the supervision of the Ministry of health as part of the national project for combating viral hepatitis. The selection and stratification of the patients were based on the availability of the kits and the financial cost of the equipment. Also, the available complete data of the patients was included in the study. Patients were subjected to thorough history taking, clinical examination and routine pre-treatment work up including: complete blood picture (CBC), serum transaminases (AST, ALT), total and conjugated bilirubin, albumin, alkaline phosphatase, urea creatinine, prothrombin time and concentration and HBsAg, HBcAb, anti-shistosomal antibodies, alpha fetoprotein (AFP), thyroid stimulating hormone (TSH), blood sugar, serum creatinine and HCV quantitative PCR. Histopathological examination of histological activity and degree of hepatic fibrosis, of ultrasound guided percutaneous liver biopsy, was performed according to the Metavir score (18). The information was largely collected from medical notes and checked for completeness and correctness. Data cleansing was applied for detecting and correcting (or removing) corrupt or inaccurate records from a record set or database in addition to the removal of typographical errors or validation and correction of values against a known list of entities. High quality data was characterized by accuracy, integrity, completeness, validity, consistency, uniformity and uniqueness.

### 3.2. Serum IL28 Quantification

Serum IL28 was assessed by enzyme-linked immunosorbent assay (ELISA) using Cusabio Biotech Co., LTD, China. This assay employs the quantitative sandwich enzyme immunoassay technique. The color development and the intensity of the color are measured.

### 3.3. DNA Collection, Extraction and IL28B Genotyping

Blood was collected into EDTA tubes. Genomic DNA was extracted using the QIAamp DNA Blood Mini Kit (Qiagen, Hilden, Germany) according to the manufacturer’s instructions. DNA quality was assessed by calculating the absorbance ratio OD260nm/280nm using NanoDrop model ND-1000 (Wilmington, USA). DNA samples were subjected to DNA quantitation and purity assessment using the NanoDrop® (ND)-1000 spectrophotometer (NanoDrop Technologies, Inc. Wilmington, USA). All equipment were calibrated. DNA quantitation the Nano-Drop spectrophotometer was blanked; a spectrum was taken as a reference material (blank) and was stored in the memory as an array of light intensity by wavelength. Measurement of a sample was taken; the intensity of light that had transmitted through the sample was recorded. The sample intensities along with the blank intensities were used to calculate the sample absorbance. The calculation of the concentration was automated. IL28B variant rs12979860 was detected using an allelic discrimination (AD) assay; a multiplexed (more than one primer/probe pair per reaction) end-point (data is collected at the end of the PCR process) assay that detects variants of a single nucleic acid sequence in TaqMan® Gene Expression Assays. The presence of two primer/probe pairs in each reaction allows genotyping of the two possible variants at the single-nucleic polymorphism (SNP) site in a target template sequence. For each sample in an AD assay, a unique pair of fluorescent dye detectors is used, for example, two TaqMan® MGB probes that target a SNP site. One fluorescent dye detector is a perfect match for the wild type (allele 1) and the other fluorescent dye detector is a perfect match for the polymorphism (allele 2).

### 3.4. Patients’ Classification

Patients were classified according to their response to pegylated interferon and ribavirin therapy into three groups. Group I: 83 non-responders (NR), including those who had detectable HCV RNA at weeks 12, 24, 48. Group II: 76 relapsers who had detectable HCV RNA 24 weeks after stoppage of treatment. Group III: 104 patients with sustained virological response (SVR) with undetectable HCV RNA six months after stopping treatment. For evaluation of the relationship between IL 28b and virus eradication, e.g. treatment success, the non-responders and relapsers were place into one group characterized by a failure to treatment (group I + group II) compared to the successful treatment group (group III).

### 3.5. Data Mining & Decision Tree Construction

Using the data mining analysis, a simple decision-tree model was constructed using Weka implementation C4.5 (19) for the likelihood prediction of SVR to interferon-ribavirin therapy. Results were classified into two groups; responders and non-responders. Decision tree analysis was carried out on the model building dataset from 263 patients using 24 variables; 3 patient characteristics (age, gender, body mass index), 4 variables from hematological tests (hemoglobin, white blood cells, ANC and platelets), 9 variables from the blood chemistry test (blood sugar, total bilirubin, indirect bilirubin, albumin, AST, ALT, alkaline phospahatase, AFP, ANA, serum level TSH) and histolopatholgical features of chronic hepatitis (grades of activity, stages of ﬁbrosis) in addition to HCV-RNA, Il 28 B polymorphism, quantitative serum IL 28 by ELISA, and type of interferon. The universality of the decision-tree model was validated using both internal and external validation to confirm the reproducibility of the results. Internal validation was performed using test mode: 10-fold cross-validation was performed which is generally applied to predict the performance of a model on a validation set using computation in place of mathematical analysis. It is a technique for assessing how the results of a statistical analysis will generalize to an independent data set. Performance of algorithms was done according to an evaluation matrix based on values for the correctly classified instance, precision, recall, F-score, and receiver operating characteristic (ROC) curve.

- Recall (sensitivity): the ability of the test to correctly identify those with the disease (true + ve rate).

- Precision (specificity): the ability of the test to correctly identify those without the disease (true - ve rate).

- F-Measure: represents the weighted average of the precision and recall.

- ROC area: area under the curve (AUC) serves as an indicator of the overall performance of the algorithm.

- Correctly classified instances which evaluate the overall accuracy.

### 3.6. Patients Consent

Informed written consent from each patient and local ethical committee approval were available before starting data collection. With respect to patients' confidentiality, patients were represented in the study by code numbers and not by their names with all personal data concealed. The study protocol conformed to the ethical guidelines of the 1975 Declaration of Helsinki.

### 3.7. Statistical Analysis

Quantitative parametric variables were presented by mean and standard deviation (SD) or inter quartile range (IQR) and compared by t-student test. Qualitative variables were compared by the chi-square or Ficher's exact test when appropriate. Receiver operator characteristic (ROC) curve was constructed to assess the level of IL28 in relation to virological response of the studied patients. Area under the curve (AUC) > 0.60 with P value < 0.05 was considered significant. Binary logistic regression analysis was done with response to treatment is the dependent factor. In all tests, p value was considered significant if < 0.05.

## 4. Results

The baseline characteristics of the 263 studied patients are shown in Table 1. The demographic features were comparable in the three groups with no statistically significant relationship between age, BMI or gender and the virological response. The mean age of the studied subjects was 44.1 ± 8.23, mean BMI was 28.42 ± 4.05, with male predominance (72.2 %). Among the baseline laboratory data AFP and AST levels were significantly lower with p values < 0.01 and 0.01 respectively and the platelets count was significantly higher with a p value of 0.015 in the SVR group than in the non-responder and relapser groups.

**Table 1.** Baseline Characteristics of the Studied Patients in Relation to Virological Response

Demographic Features	Non Responder	Relapsers	Responders	P value
**Gender No. (%)**				0.21
Male 190 (72.2%)	54 (28.4)	57 (30)	79 (41.6)	
**Female 73 (27.8%)**	29 (40)	19 (26)	25 (34)	
**Age, y, Mean ± SD**	44.78 ± 8.1	45.27 ± 7.82	42.94 ± 8.23	0.207
**BMI^a^, Mean ± SD**	28.9 ± 4.47	27.94 ± 3.63	28.42 ± 4.07	0.378
**Baseline Laboratory Data**				
Hemoglobin (12-16g/dl), Mean ± SD	13.93 ± 1.66	14.18 ± 1.53	14.36 ± 1.56	0.4
TLC^a^(4.000-11.000/mm^3^), Mean ± SD	6.01 ± 1.6	6.76 ± 2.23	6.82 ± 1.79	0.76
ANC^a^, Mean ± SD	3.1 ±1.09	3.82 ±1.97	3.55 ±1.35	0.12
Platelets (150.000-400.000/mm^3^), Mean ± SD	197.7 ± 66.7	203.3 ± 64.03	231.1 ± 25.3	0.015
ALT^a^(40) IU/L, Mean ± SD^b^	56 ± 51	52 ± 44	52 ± 42	0.23
AST^a^(40) IU/L, Mean ± SD^b^	53 ± 46	49 ± 38	45 ± 33	0.01
Bilirubin (0.3-1.2mg/dl), Mean ± SD	0.51 ± 0.19	0.56 ± 0.27	0.56 ± 0.24	0.56
ALP^a^(290) IU/L^b^, Mean ± SD	151 ± 124	161 ± 129	151 ± 120	0.81
Albumin (3.5-5g/dl), Mean ± SD	4.29 ± 0.42	4.15 ± 0.46	4.32 ± 0.48	0.89
Creatinine (mg/dl), Mean ± SD	0.87 ± 0.17	0.90 ± 0.20	0.90 ± 0.30	0.214
Glucose (70-130 mg/dl), Mean ± SD	102.2 ± 28.2	102.7 ± 27.7	100.4 ± 22.3	0.87
TSH^a^(0.5-4.5 mIU/L), Mean ± SD	1.69 ± 1.28	1.39 ± 0.81	1.52 ± 0.94	0.34
AFP^a^(10 ng/dl)^b^, Mean ± SD	4 ± 7.4	5.1 ± 6.1	2.3 ± 2.9	0.001
Hepatitis C Virus RNA log (10 IU)^b^, Mean ± SD	5.14 ± 1.12	5.04 ± 1.14	5.02 ± 1.27	0.86
**Histological Parameters**				
< F^a^2 N = 170 (64.4%), No. (%)	52 (30.5)	47 (27.6)	71 (41.9)	0.45
≥ F2 N = 93 (35.4%), No. (%)	31 (33.3)	29 (31.2)	33 (35.5)	
< A^a^2 N = 234 (89.1%), No. (%)	74 (31.6)	67 (28.6)	93 (39.8)	0.76
≥ A2 N = 29 (10.9%), No. (%)	9 (31)	9 (31)	11 (38)	

^a^Abbreviations: BMI, body mass index; TLC, total leucocytic count; ANC, absolute neutrophilic count; AST, aspartate aminotransferase; ALT, alanine aminotransferase; ALP, alkaline phosphatase; TSH, Thyroid stimulating hormone; AFP, alpha fetoprotein; F, fibrosis; A, activity

^b^Median (IQR) Mann-Whitney u test

**Figure 1.**
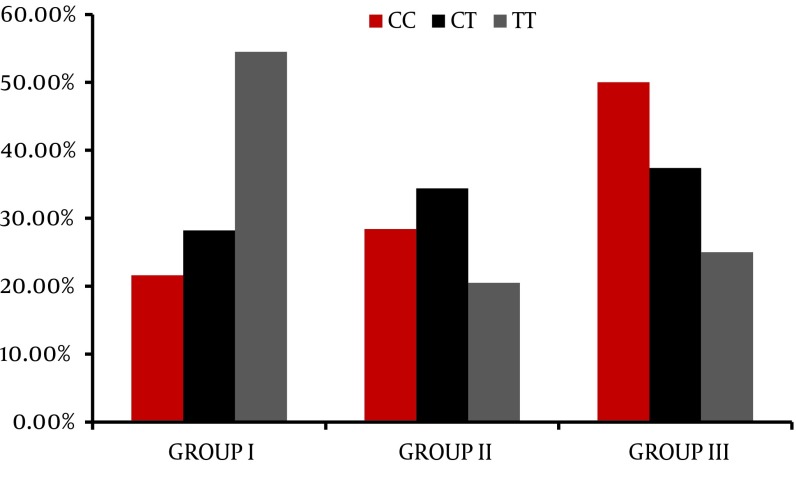
Il28 B Genotype Frequency in Relation to Virological Response in the Studied Groups

**Figure 2.**
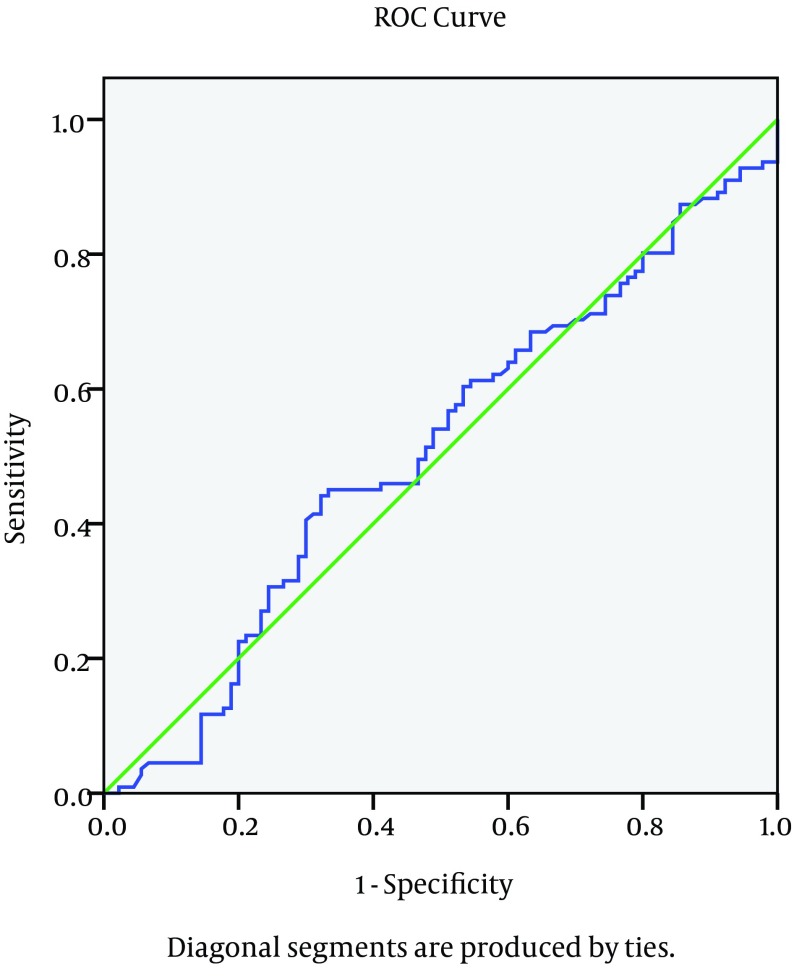
ROC Curve for Serum IL28 Level in Relation to Sustained Virological Response of Treatment

The genotype distribution for IL28 B polymorphism is different between the different groups; the absence of C allele (e.g. TT genotype) is significantly associated with early failure of response as presented in the NR group with p value = 0.01, while the CC is significantly correlated with favorable response as presented in the SVR group with p value 0.03 as plotted in Figure 1. The relationship between serum IL28 expression and virological response to treatment showed that there is no good reliable cut off for serum IL 28 B to predict the response of treatment as shown in the ROC curve in Figure 2. There was no relationship between serum IL 28 B and different IL28 B genotypes; CC, CT and TT genotype serum levels were 34.8 ± 39.9, 28.7 ± 31.5 and 33.4 ± 25.4, respectively. Correlation between IL28 B and the significant baseline laboratory parameters with the response (AFP, AST and platelets) is shown in Table 2, with no statistically significant correlation between IL28B and any of the mentioned parameters. In relation to the histopathology of the studied patients, there were no statistically significant relationship found between IL28 genotype and the different degrees of inflammation and stages of fibrosis in our studied patients with P value 0.31 and 0.4 respectively.

**Table 2.** Non-Parametric Correlation Between IL28B and AFP, AST and Platelets

IL28B	Spearman correlation	P value
**AFP^a^**	-0.081	0.37
**AST^a^**	0.008	0.92
**Platelets**	0.044	0.622

^a^Abbreviations: AFP, alpha fetoprotein; AST, aspartate aminotransferase

**Figure 3.**
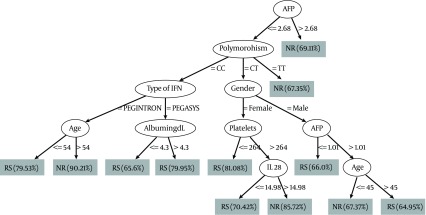
Decision Tree Analysis of the 263 Studied Patients

Baseline level of AFP at a cutoff level of ≤ 2.68ng/ml was selected as the variable of initial split, with 69.11 % of non-responder patients with AFP level > 2.68 ng/ml. Whereas in patients with AFP level < 2.68 ng/ml, Il 28 B polymorphism was selected as the variable of second split B where the patients with TT genotype had the highest probability of failure of response (67.35 %). Combination of other variables such as serum albumin, age, platelets, type of interferon, gender and serum IL 28 B were shown to have some role in prediction as seen in Figure 3. The relationship between AFP, IL 28 B polymorphism and response is shown in Table 3 ; we used the variable of the first split in the decision tree which is the AFP with cutoff 2.68 ng/ml and the variable of the second split IL 28 B polymorphism in relation to response. Results showed that in the CC genotype, patients with AFP below the mentioned cutoff show better response while those with AFP value above the cutoff showed poor response with P value 0.02, and the same occurred for the CT genotype with p value 0.01, while in the TT genotype AFP failed to predict the outcome with its previously mentioned cutoff. Only variables that showed significance in decision tree was entered in univariate analysis; the variables that achieved P < 0.25 only further passed to multivariate analysis. In multivariate logistic regression, in which treatment response is the dependent factor, AFP level more than 2.68ng/ml and platelets below 224.000/mm3 are the independent factors that was associated with failure of outcome; the results of the analysis are shown in Table 4.

**Table 3.** AFP in Relation to Response and IL 28 B Polymorphism

		SVR, No. (%)	Relapser + NR, No. (%)	Total, No. (%)	P value
**CC**	< 2.68	14 (70)	6 (30)	20 (100)	0.02
	˃ 2.68	7 (33.3)	14 (66.7)	21 (100)	
**CT**	< 2.68	16 (61.5)	10 (38.5)	26 (100)	0.01
	˃ 2.68	18 (32.1)	38 (67.9)	56 (100)	
**TT**	< 2.68	2 (33.3)	4 (66.7)	6 (100)	1
	˃ 2.68	3 (27.3)	8 (72.7)	11 (100)	

**Table 4.** Multivariate Logistic Regression Analysis in Relation to Failure of Treatment as Dependent Factor

Independent factor	Odd’s Ratio	95% CI	P value
**AFP^a^(> 2.68 ng/ml)**	2.96	2.16-3.76	0.007
**Platelets (< 224.000 /mm^3^)**	0.993	0.98-1.0	0.031
**AST^a^(> 40 IU/l)**	1.0	0.994-1.00	0.63

^a^Abbreviations: AFP, alpha fetoprotein; AST, aspartate aminotransferase

## 5. Discussion

Hepatitis C virus (HCV) genotype 4 is the most frequent cause of chronic hepatitis C in the Middle East, North Africa, and sub-Saharan Africa (20). Various studies from European and Middle Eastern countries showed that the sustained virological response in genotype 4 for combination therapy, pegylated interferon and ribavirin, ranges between 43%-70% (21-23). Histopathological assessment of the grade of activity and degree of fibrosis of the studied groups shows no statistically significant relationship between either activity or fibrosis and the virological response. The frequency of the IL28 B genotype showed that more than half of the studied population is CT (56 %), followed by CC (25%) then TT (19%). Little is known about predictors of response within populations infected with genotype 4. In previous studies on genotype 4; age, pretreatment viral load, and stage of fibrosis were considered as good predictive factors (24, 25).

The gene expression and the role of IL28B gene SNP rs12979860 in response to treatment in genotype 4 were recently studied by limited research with CC genotype of higher response rate (26, 27). The objective of this study was to evaluate the expression and the predictive power of the rs12979860 IL28B SNP and its protein for treatment response in HCV-4 Egyptian patients by regression analysis and decision tree analysis in relation to other predictors of response.

Relationship between baseline parameters and virological response showed that, lower base line AST, AFP level and higher platelet count are significantly associated with favorable outcomes.

In our study, lower base line AST but not ALT was an independent predictor of SVR in patients with chronic HCV genotype 4. This was in accordance with previous studies which demonstrated that the AST reflects less severe histopathological parameters in sustained responders (28). Higher serum AFP level was the strongest predictor of failure to achieve SVR in the studied patients. Previous studies including HCV genotype 4 (29, 30) and genotype 1 (31, 32) highlighted the same findings. Abdoul et al., 2008 examined the association between serum alpha-fetoprotein (AFP) level and sustained virological response (SVR) in 93 chronic hepatitis C patients and found that the SVR rate was much higher among patients with serum AFP levels below rather than above a median value of 5.7 ng/ml, denoting that serum AFP should be added to the list of factors predictive of treatment response in chronic hepatitis C (33). Low platelets count was associated with lower SVR level. This may be because lower platelet count is a hallmark of advanced fibrosis in chronic hepatitis C and has been reported to be associated with poor response to IFN (34, 35). Cielsa et al., in 2012 showed that assessment of the platelet level and the IL28B polymorphism can complement the decision-making algorithm for a patient’s eligibility for antiviral therapy (36). The IL28B polymorphism rs12979860 has a marked differential distribution between racial groups, being least frequent in African Americans, most frequent in Asians, and with an intermediate frequency in Hispanics and Caucasians (10, 37). The frequency of IL28 genotype in our genotype 4 Egyptian patients showed that almost half of them were of the CT genotype (56 %) followed by CC (25 %) while TT had the least expression (19 %). Di Nicola group which included 128 patients with genotype 4, 68% Egyptians, showed 63 % CT, 14 % TT, and 23 % CC expression (27). Also, Asselah and colleagues studied 164 patients with genotype 4 (43% Egyptians), and found the difference in distribution of IL 28 B genotype between Egyptians and Subsaharan Africans; in the Egyptian ethinicity the frequency was 55% CC, 11% TT and 34% CT, while in the in sub-Saharan group the TT genotype was the most predominant form (48 %) (26). El-Awady and colleagues during 2012 also in a study on genotype 4, found that the frequencies of genotypes were 48% CC, 14% TT, and 38% CT for their studied patients (38). In the current study, the CC genotype was significantly correlated with SVR in comparison to CT and CC. The response rates were 50%, 47.4% and 25% for genotype CC, CT, and TT respectively. Absence of C allele (TT genotype) was associated with 75% failure of response; either early failure, e.g. non response (54.5%), or late failure, e.g. relapsers (20.5 %). This is in agreement with the previous studies reported on genotype 1 (12, 36, 39) and studies conducted on genotype 4 (26, 27). No relationship was noticed between the serum level of Il-28 and the different genotypes in the studied patients. However, in a previous study, serum IL-29 and IL-28 corresponded with TT genotype irrespective of the treatment response (14). There was no statistically significant relationship between IL28 B genotype and the degree of activity and stage of fibrosis in our studied patients. Previous studies found that the IL28B locus is not associated with the risk of developing advanced fibrosis (26, 40). However, previous studies reported that TT genotype occurred more frequently in patients with end stage liver disease (36, 38, 41). Unfortunately, we didn’t find a significant correlation between IL28 and independent predictors of response (AFP, AST and Platelets). In contrary to other studies done on HCV genotype 4 were Il28 B has a place in the treatment algorithm in HCV genotype 4 patients (26, 27). In countries with high prevalence for HCV such as Egypt, new insight into HCV-4 infection may result in a refinement of the treatment strategies to individualize therapies to reduce the unfavorable implications in terms of cost and tolerability (42). The data mining methodology and decision-tree analysis were used to construct a simple decision tree model using the readily-available standard tests together with IL28 B genotype and serum IL-28 to predict SVR with high-probability (16, 43). Using this model, rapid estimates of the response before treatment can be made by allocating patients to specific subgroups with a defined rate of response simply by following the flowchart form. Our results were able to identify previously unnoticed, close associations between baseline AFP levels and the likelihood of response in chronic HCV patients. A rather interesting finding was that the low serum AFP (< 2.68 ng/ml) was the first split variable in the predictive model for response and was significantly associated with SVR in the multivariate analysis as well. Also, the TT genotype had the highest probability of failure of response (67.35 %) in the decision tree model and up to 75% on statistical analysis. We used the results in our decision tree to find the relationship between different IL 28 B genotypes and AFP cutoff (2.68 ng/ml). We found that in CC, CT genotype patients below 2.68 ng/ml are more likely to respond to treatment than those above the mentioned cutoff. While in TT genotype the AFP failed to predict the treatment outcome by its mentioned cutoff.

To conclude, baseline AFP is an important predictor of antiviral therapy response in chronic HCV, with suggested cut off level of < 2.68 ng/ml. Absence of C allele (TT genotype) is less likely to respond to the current standard of care therapy interferon-ribavirin therapy. Data mining analysis and decision-tree model can be used as good prognostic algorithms that could be beneficial in segregating patients according to likely clinical outcomes to guide clinical management.
